# Oncological impact of inflammatory biomarkers in elderly patients treated with radical cystectomy for urothelial bladder cancer

**DOI:** 10.1080/2090598X.2020.1814974

**Published:** 2020-08-26

**Authors:** Andrea Mari, Gianluca Muto, Fabrizio Di Maida, Riccardo Tellini, Riccardo Bossa, Claudio Bisegna, Riccardo Campi, Andrea Cocci, Lorenzo Viola, Antonio Grosso, Sabino Scelzi, Alberto Lapini, Marco Carini, Andrea Minervini

**Affiliations:** a Unit of Oncologic Minimally-Invasive Urology and Andrology, Careggi Hospital, Florence, Italy; b Department of Experimental and Clinical Medicine, University of Florence, Florence, Italy

**Keywords:** Urothelial bladder cancer, radical cystectomy, biomarkers, systemic inflammation, recurrence, mortality

## Abstract

**Objective:**

To evaluate the impact of preoperative markers of systemic inflammation on complications and oncological outcomes in patients aged ≥75 years treated with radical cystectomy (RC) for urothelial bladder cancer (UBC).

**Patients and methods:**

The clinical data of 694 patients treated with open RC for UBC at our institution between January 2008 and December 2015 were retrospectively reviewed. Patients aged <75 years, with distant metastases, other-than-urothelial histological type, comorbidities that could affect the systemic inflammatory markers, and patients who received neoadjuvant chemotherapy were excluded. Multivariable regression models were built for the prediction of major postoperative surgical complications, disease recurrence, cancer-specific mortality (CSM), and overall mortality (OM).

**Results:**

The median (interquartile range [IQR]) age at surgery was 79 (75–83) years. Major postoperative surgical complications were registered in 41.9% of the patients. The 5-year overall survival, cancer-specific survival and recurrence-free survival rates were 42.4% (95% confidence interval [CI] 34.7–49.9%), 70.3% (95% CI 62.3–76.9%), and 59.8% (95% CI 52.4–66.5), respectively. At multivariable analysis, higher levels of fibrinogen and a modified Glasgow Prognostic Score (mGPS) of 1 and 2 at baseline were independently associated with higher risk of major postoperative complications and of CSM. The inclusion of mGPS and fibrinogen to a standard multivariable model for recurrence and for CSM increased discrimination from 69.4% to 73.0% and from 71.3% to 73.9%, respectively. Preoperative neutrophil-to-lymphocyte ratio of >3 was independently associated with OM (hazard ratio 1.38, 95% CI 1.01–1.77; *P* = 0.01).

**Conclusions:**

In a cohort of elderly patients with UBC treated with RC, fibrinogen and mGPS appeared to be the most relevant prognostic measurements and increased the accuracy of clinicopathological preoperative models to predict major postoperative complications, disease recurrence and mortality.

**Abbreviations:**

ASA: American Society of Anesthesiologists; CCI: Charlson Comorbidity Index; CIS: carcinoma *in situ*; CRP: C-reactive protein; CSM: cancer-specific mortality; CSS: cancer-specific survival; ECOG PS: Eastern Cooperative Oncology Group Performance Status; HDL: high-density lipoprotein; (S)HR: (subdistribution) hazard ratio; LND: lymphadenectomy; LVI: lymphovascular invasion; mGPS: modified Glasgow Prognostic Score; NLR: neutrophil-to-lymphocyte ratio; NOC: non-organ-confined; OM: overall mortality; OR: odds ratio; OS: overall survival; RC: radical cystectomy; RNU: radical nephroureterectomy; UBC: urothelial bladder cancer; UTUC: upper urinary tract urothelial carcinoma

## Introduction

Radical cystectomy (RC) for the treatment of urothelial bladder cancer (UBC) is burdened by a non-negligible rate of postoperative morbidity and mortality despite the technological and surgical technique improvements in recent years [1]. Indeed, urologists are often faced with frail and elderly patients presenting at diagnosis with poor performance status and specifically needing a careful assessment of advantages and risks while deciding the most suitable treatment option [2–4].

Numerous clinical prognostic models have been proposed to evaluate the risk of recurrence or mortality in patients with UBC amenable to RC. Therefore, besides the multitude of clinical, radiological, genetic and pathological variables, serum markers of systemic inflammation may be particularly useful, not only for their intrinsic prognostic value, but also for the ease of accessibility, the lack of invasiveness and the cost-effectiveness of their assessment [5]. The tumour microenvironment induces the production and the release of cytokines that modify the systemic inflammatory response playing a crucial role in the development and progression of the disease [6]. Pretreatment measurements of inflammatory markers such as lymphocytes, neutrophils, high-density lipoprotein (HDL), albumin and the C-reactive protein (CRP) have been combined in a variety of scores, indices, and ratios, including the neutrophil-to-lymphocyte ratio (NLR) and the modified Glasgow Prognostic Score (mGPS). Several series showed that the accuracy of prognostic models increased when these markers are added to the clinicopathological features [7,8]. Nevertheless, data regarding the impact of these biomarkers has never been applied to a selected population of elderly patients. Moreover, the role of preoperative plasma fibrinogen has not been fully investigated in predicting the oncological outcome in patients with UBC [9].

To address these unmet needs, we sought to analyse the clinical data of elderly patients treated with open RC at our Institution to evaluate the role of preoperative fibrinogen, mGPS, NLR, HDL and total cholesterol on major postoperative complications and long-term oncological outcomes.

## Patients and methods

After Institutional Review Board approval, we reviewed our prospectively collected database to analyse clinical and surgical data from 694 patients treated at our Institution with open RC for UBC between January 2008 and December 2015. Of these, 347 elderly patients (aged ≥75 years) were selected for the current study. We excluded patients with distant metastases at baseline (11 patients), histological types other than UCB (nine), patients who received neoadjuvant chemotherapy (19), patients with comorbidities that could affect the systemic inflammatory markers (i.e. immunological and haematological disorders, chronic liver disease, autoimmune diseases, and chronic inflammatory diseases, nine), and patients without all the serum inflammatory markers available at baseline (44).

The preoperative evaluation included chest CT and contrast-enhanced CT or MRI of the abdomen. Clinical, surgical and pathological features were recorded. Preoperative performance status was estimated by using Charlson Comorbidity Index (CCI), Eastern Cooperative Oncology Group Performance Status (ECOG PS) Scale, American Society of Anesthesiologists (ASA) physical classification system and chronic kidney diseases status. TNM was reported according to the American Joint Committee on Cancer (AJCC) Staging Manual, eighth edition [10]. Lymphovascular invasion (LVI) was defined as the presence of tumour cells within an endothelium-lined space [11,12].

Serum albumin levels were estimated by the bromocresol green albumin method. Plasma fibrinogen levels were measured by the Clauss standard method with bovine thrombin. The mGPS was calculated as follows: patients with elevated CRP serum levels (>10 mg/L) and hypoalbuminaemia (<3.5 g/dL) were allocated a score of 2, and patients with one or no abnormal value were allocated a score of 1 or 0, respectively. The NLR threshold value (NLR >3) was based on the most frequent value analysed in the literature [7,8].

Surgery was performed by four experienced surgeons who usually perform individually >30 RCs/year. The procedure was performed according to the traditional open technique, lymphadenectomy (LND) was performed in 80.4% of the patients. Standard LND included the removal of presacral, internal iliac, obturator fossa and external iliac nodes, with the ureter and genitofemoral nerves being respectively the medial and lateral borders. Caudally it was limited by the circumflex iliac vein, the lacunar ligament and the lymph node of Cloquet. As appropriate, if further lymph nodes were found clinically suspicious for tumour involvement, either an extended or super-extended LND was performed as follows: during the extended LND, nodes were removed in the region of the aortic bifurcation, presacral and common iliac vessels medial to the crossing ureter, whereas it extended cranially to the level of the inferior mesenteric artery when super-extended LND was necessary.

### Follow-up and outcomes

Surgical postoperative complications were defined as any postoperative event caused by surgery until the third postoperative month, altering the normal postoperative course and/or delaying discharge. The severity of complications was graded according to the modified Clavien–Dindo classification. Major postoperative complications were defined as those complications graded as ≥III. The follow-up schedule included blood analysis, chest and abdomen CT scan every 6 months until the third postoperative year, followed by annual imaging thereafter according to the current International guidelines [13].

The outcomes explored were the major postoperative complications, recurrence, cancer-specific mortality (CSM) and overall mortality (OM).

### Statistical analysis

First, descriptive statistics were obtained reporting median (interquartile range [IQR]) for continuous variables, and frequencies and proportions for categorical variables, as appropriate. Second, univariate and multivariable logistic regressions for postoperative major surgical complications were performed. Third, a univariable Cox proportional hazards regression was performed to explore the impact of clinicopathological feature and biomarkers on recurrence, CSM and OM. A multivariable competing risk analysis was carried out for recurrence and CSM to exclude the bias of premature death due to other causes considering the elderly population. A multivariable Cox regression was performed for OM. Fourth, discrimination of multivariable models was evaluated using the Harrell concordance index.

Statistical significance was set as *P* < 0.05. All tests were two-sided. Analyses were carried out using STATA, version 14.1 (StataCorp LP, College Station, TX, USA).

## Results

### Descriptive analysis

Table 1 summarises the baseline clinical and demographic characteristics of the patients included in the study. The median (IQR) age at surgery was 79 (75–83) years and 212 (83.1%) were males. The median (IQR) preoperative plasma fibrinogen, HDL and total cholesterol level were respectively 361 (299–442), 49 (43–57) and 221 (187–263) mg/dL. The mGPS was graded 2 in 48 (18.8%) patients, 1 in 82 (32.2%) patients, and 0 in 125 (49%) patients. Preoperative hydronephrosis was registered in 25.9% of cases. The median (IQR) preoperative NLR was 2.37 (1.61–3.53).

**Table 1.** Descriptive analysis of the preoperative characteristics of 255 elderly patients treated with RC for UBC

Variable	Value
Number of patients	255
Sex, *n* (%)	
Male	212 (83.1)
Female	43 (16.9)
Smoking, *n* (%)	
Never	66 (25.8)
Former	97 (38.1)
Current	92 (36.1)
Smoking intensity, *n* (%)	
Never	66 (25.8)
High	74 (29.1)
Low	115 (45.1)
Age at UBC diagnosis, years, median (IQR)	78 (74–82)
Age at RC, years, median (IQR)	79 (75–83)
Preoperative BMI, kg/m^2^, median (IQR)	23.4 (20.4–28.3)
Previous radiotherapy treatment, *n* (%)	
No	251 (98.4)
Yes	4 (1.6)
ECOG PS score, *n* (%)	
0	139 (54.5)
1	103 (40.4)
2	13 (5.1)
ASA score, median (IQR)	3 (2–4)
CCI score, median (IQR)	3 (2–4)
Clinical T stage, *n* (%)	
T1	12 (4.7)
T2	170 (67.2)
T3	56 (22.1)
T4	15 (5.9)
Clinical N stage, *n* (%)	
N0	241 (94.5)
N+	14 (5.5)
Preoperative haemoglobin, g/dL, median (IQR)	13.2 (11.7–14.5)
Preoperative albumin, g/dL, median (IQR)	4.2 (3.9–4.4)
Preoperative CRP, mg/L, median (IQR)	8.8 (6.7–11.5)
mGPS, *n* (%)	
0	125 (49.0)
1	82 (32.2)
2	48 (18.8)
Hydronephrosis, *n* (%)	
No	189 (74.1)
Yes	66 (25.9)
Preoperative eGFR, mL/min/1.73 m^2^, median (IQR)	69.70 (49.27–89.70)
Preoperative fibrinogen level, mg/dL, median (IQR)	361 (299–442)
Preoperative NLR, median (IQR)	2.37 (1.61–3.53)
Preoperative total cholesterol level, mg/dL, median (IQR)	221 (187–263)
Preoperative HDL level, mg/dL, median (IQR)	49 (43–57)

Pathological and perioperative features are described in Table 2. A non-organ-confined (NOC) disease was found in 173 (67.9%) patients, of whom 117 (45.9%) were pT3 and 56 (22%) were pT4. The lymph nodes were positive in 37.1% of cases, while LVI was present in 71 (27.8%) patients. Overall, surgical postoperative complications occurred in 107 (41.9%) patients, of which 14% were major. The postoperative mortality rate at ≤3 months from surgery was 2.3%. The 5-year overall survival (OS) was 42.4% (95% CI 34.7–49.9%), the 5-year cancer-specific survival (CSS) was 70.3% (95% CI 62.3–76.9%), and the recurrence-free survival at 5 years was 59.8% (95% CI 52.4–66.5%).

**Table 2.** Descriptive analysis of the intra- and postoperative characteristics of 255 elderly patients treated with RC for UBC

Variable		Value
Year of cystectomy, *n* (%)		
2008		23 (9.0)
2009		14 (5.5)
2010		14 (5.5)
2011		18 (7.1)
2012		30 (11.8)
2013		16 (6.3)
2014		43 (16.9)
2015		97 (38.0)
Extension of LND		
Not performed		50 (19.6)
Limited		141 (55.3)
Extended		49 (19.2)
Super-extended		15 (5.9)
Overall LNs removed, *n*, median (IQR)		13 (7–21)
Overall positive LNs, *n* (%) 0 1		157 (61.6) 35 (13.7)
2		18 (7.1)
>2		45 (17.6)
Pathological T-stage at RC, *n* (%)		
T1		15 (5.9)
T2		67 (26.3)
T3		117 (45.9)
T4		56 (22.0)
Pathological N-stage at RC, *n* (%)		
Nx		49 (18.6)
N0		108 (40.9)
N1		35 (13.3)
N2		36 (13.6)
N3		27 (10.2)
Concomitant CIS, *n* (%)		98 (38.4)
Concomitant LVI, *n* (%)		71 (27.8)
Overall postoperative complications, *n* (%)		107 (41.9)
Clavien–Dindo Grade I, *n* (%)		52 (20.4)
Clavien–Dindo Grade II, *n* (%)		19 (7.5)
Major	Clavien–Dindo Grade IIIa, *n* (%)	11 (4.3)
	Clavien–Dindo Grade IIIb, *n* (%)	10 (3.9)
	Clavien–Dindo Grade IV, *n* (%)	7 (2.7)
	Clavien–Dindo Grade V, *n* (%)	8 (3.1)

### Factors predicting major postoperative complications

Table 3 shows the uni- and multivariable logistic regression for postoperative major surgical complications. At multivariable analysis, ASA score (odds ratio [OR] 1.42, 95% CI 1.19–1.70; *P* < 0.001), age at RC (OR 1.05, 95% CI 1.02–1.07; *P* = 0.01), lower haemoglobin (OR 0.98, 95% CI 0.95–0.99; *P* = 0.01) and fibrinogen (OR 1.005, 95% CI 1.003–1.013; *P* = 0.001) at baseline, mGPS 1 (OR 1.74, 95% CI 1.31–2.30; *P* < 0.001) and 2 (OR 2.44, 95% CI 1.55–3.82; *P* < 0.001) compared to 0, a NOC (OR 2.55, 95% CI 1.40–4.61; *P* = 0.001) and pathological N positive (OR 2.58, 95% CI 1.46–4.53; *P* = 0.001) disease were independently associated with major postoperative complications.

**Table 3.** Uni- and multivariable analysis for major surgical complications in 255 elderly patients treated with RC for UBC

Predictive analysis for surgical major complications		Univariable analysis		Multivariable analysis	
		OR (95%CI)	*P*	OR (95%CI)	*P*
ASA score		1.37 (1.14–1.67)	<0.001	1.42 (1.19–1.70	<0.001
ECOG PS score		1.43 (1.09–1.88)	0.01	1.27 (0.89–1.79	0.19
Age at RC		1.06 (1.02–1.10)	0.001	1.05 (1.02–1.07	0.001
CCI score		1.08 (0.96–1.20)	0.20	-	-
Preoperative hydronephrosis		1.36 (0.65–2.94)	0.46	-	-
Preoperative haemoglobin		0.97 (0.94–0.99)	0.01	0.98 (0.95–0.99	0.01
Preoperative creatinine		1.01 (0.98–1.03)	0.43	-	-
Preoperative eGFR		0.99 (0.99–1.00)	0.96	-	-
Preoperative BMI		1.02 (0.99–1.04)	0.14	-	-
Preoperative total cholesterol		1.01 (0.97–1.04)	0.52	-	-
Preoperative HDL		0.99 (0.96–1.01)	0.49	-	-
NLR >3		1.55 (1.12–2.13)	0.01	1.19 (0.97–1.45	0.09
Preoperative fibrinogen		1.009 (1.003–1.013)	0.001	1.005 (1.003–1.013	0.001
Preoperative albumin		0.62 (0.47–0.82)	0.001	-	-
Preoperative CRP		1.22 (1.11–1.32)	<0.001	-	-
mGPS	1 vs 0	1.76 (1.32–2.34)	<0.001	1.74 (1.31–2.30	<0.001
	2 vs 0	2.41 (1.54–3.75)	<0.001	2.44 (1.55–3.82	<0.001
pT3–4		2.13 (1.19–3.80)	0.01	2.55 (1.40–4.61	0.001
pN+		2.89 (1.53–5.42)	0.001	2.58 (1.46–4.53	0.001
Concomitant CIS at RC specimen		1.42 (0.63–3.16)	0.39	-	-
LVI at RC specimen		1.09 (0.59–1.99)	0.78	-	-

### Factors predicting survival outcomes

Tables 4 and 5 show the uni- and multivariable analysis predicting disease recurrence, CSM and OM.

**Table 4.** Univariable analysis of disease recurrence, CSM and OM in 255 elderly patients treated with RC for UBC

		Recurrence		CSM		OM	
		Cox regression		Cox regression		Cox regression	
Univariable analysis		HR (95% CI)	*P*	HR (95% CI	*P*	HR (95% CI)	*P*
ASA score		0.84 (0.38–1.23)	0.38	1.38 (0.83–2.28)	0.21	1.04 (0.74–1.47)	0.78
ECOG PS score		1.39 (0.99–1.95)	0.06	1.39 (0.93–2.08)	0.10	1.46 (1.10–1.94)	0.008
Age at RC		1.02 (0.99–1.05)	0.11	1.05 (1.01–1.09)	0.01	1.09 (1.06–1.12)	<0.001
CCI score		1.02 (0.89–1.18)	0.72	0.95 (0.78–1.45)	0.59	0.92 (0.80–1.05)	0.24
Preoperative hydronephrosis		1.002 (0.87–1.15)	0.97	1.06 (0.64–1.78)	0.80	1.21 (0.98–1.93)	0.08
Preoperative haemoglobin		1.10 (0.71–1.71)	0.67	0.94 (0.81–1.10)	0.49	0.95 (0.85–1.06)	0.41
Preoperative eGFR		0.99 (0.99–1.00)	0.97	1.00 (0.99–1.01)	0.30	0.99 (0.99–1.01)	0.06
Preoperative BMI		1.01 (0.97–1.06)	0.60	1.01 (0.96–1.06)	0.72	1.02 (1.00–1.03)	0.05
Preoperative total cholesterol		1.00 (0.99–1.00)	0.76	1.00 (0.99–1.00)	0.63	1.00 (0.99–100)	0.81
Preoperative HDL		0.99 (0.97–1.01)	0.36	0.98 (0.96–1.01)	0.13	0.99 (0.97–1.01)	0.39
NLR (>3 vs ≤3)		1.36 (1.07–1.71)	0.01	1.59 (1.18–2.13)	0.002	1.34 (1.01–1.77)	0.04
Preoperative fibrinogen		1.009 (1.005–1.012)	<0.001	1.007 (1.003–1.010)	0.02	1.003 (0.998–1.009)	0.034
Preoperative albumin		0.64 (0.39–0.99)	0.045	0.48 (0.27–0.68)	0.001	0.65 (0.42–0.89)	0.001
Preoperative CRP (mg/L)		1.23 (1.15–1.32)	<0.001	1.19 (1.10–1.30)	<0.001	1.05 (0.99–1.10)	0.08
mGPS	1 vs 0	3.14 (1.78–5.33)	<0.001	3.53 (2.11–9.51)	<0.001	1.12 (0.71–1.78)	0.61
	2 vs 0	4.40 (4.33–12.57)	<0.001	4.73 (2.19–10.12)	<0.001	1.68 (1.11–2.54)	1.78
pT3–4 vs pT1–2		5.51 (4.26–8.67)	<0.001	4.27 (2.39–5.76)	<0.001	0.90 (0.61–1.34)	0.62
pN+		1.71 (1.12–2.62)	0.01	2.32 (1.75–3.44)	<0.001	2.11 (1.32–2.81)	0.004
Concomitant CIS at RC specimen		1.34 (0.93–1.91)	0.11	1.34 (0.93–1.91)	0.11	1.34 (0.93–1.91)	0.11
LVI at RC specimen		1.87 (1.17–3.02)	0.02	1.65 (1.02–2.66)	0.04	1.29 (0.82–2.02)	0.26

**Table 5.** Multivariable analysis for disease recurrence, CSM and OM in 255 elderly patients treated with RC for UBC

		Recurrence		CSM		OM	
		Competing-risk regression		Competing-risk regression		Cox regression	
Multivariable analysis		SHR (95% CI)	*P*	SHR (95% CI)	*P*	HR (95% CI)	*P*
ASA score		-	-	-	-	-	-
ECOG PS score		-	-	1.26 (0.79–2.00)	0.33	1.18 (0.75–1.87)	0.46
Age at RC		1.02 (0.99–1.04)	0.23	-	-	1.04 (1.01–1.08)	0.001
CCI score		-	-	-	-	-	-
Preoperative hydronephrosis		-	-	-	-	-	-
Preoperative haemoglobin		-	-	-	-	-	-
Preoperative creatinine		-	-	-	-	-	-
Preoperative eGFR		-	-	-	-	-	-
Preoperative BMI		-	-	-	-	-	-
Preoperative total cholesterol		-	-	-	-	-	-
Preoperative HDL		-	-	-	-	-	-
NLR >3		1.13 (0.96–1.32)	0.14	1.21(0.99–1.48)	0.06	1.38 (1.01–1.77)	0.01
Preoperative fibrinogen		1.005 (1.002–1.009)	0.001	1.003 (1.001–1.008)	0.001	-	-
Preoperative albumin		-	-	-	-	0.63 (0.49–0.92)	0.001
Preoperative CRP (mg/L)		-	-	-	-	-	-
mGPS	1 vs 0	2.96 (1.72–6.28)	0.01	2.26 (2.16–5.82)	0.001	-	-
	2 vs 0	2.48 (2.50–7.35)	0.01	2.44 (2.37–6.13)	0.001	-	-
pT3–4 vs pT1–2		4.58 (2.61–9.37)	0.001	2.57 (1.39–5.76)	<0.001	1.49 (1.19–5.76)	<0.001
pN+		3.52 (2.46–6.27)	0.004	3.36 (1.76–3.45)	<0.001	3.36 (1.75–3.44)	<0.001
Concomitant CIS at RC specimen		-	-	-	-	-	-
LVI at RC specimen		1.78 (1.06–2.99)	0.03	1.48 (0.99–2.17)	0.05	-	-
*C-index*		*0.734*		*0.739*		*0.712*	

At multivariable analysis, baseline fibrinogen (subdistribution hazard ratio [SHR] 1.005, 95% CI 1.002–1.009; *P* = 0.001), mGPS 1 (SHR 2.96, 95% CI 1.72–6.28; *P* = 0.01) and 2 (SHR 2.48, 95% CI 2.50–7.35; *P* = 0.01) compared to 0, NOC disease (SHR 4.58, 95% CI 2.61–9.37; *P* = 0.001), pathological N positive disease (SHR 3.52, 95% CI 2.46–6.27; *P* = 0.004) and LVI at RC specimen (SHR 1.78, 95% Cl 1.06–2.99; *P* = 0.03) were independently associated with disease recurrence.

Moreover, preoperative fibrinogen (SHR 1.003, 95% CI 1.001–1.008; *P* = 0.001), mGPS 1 (SHR 2.26, 95% CI 2.16–5.82; *P* = 0.001) and 2 (SHR 2.44, 95% CI 2.37–6.13; *P* = 0.001) compared to 0, NOC disease (SHR 2.57, 95% CI 1.39–5.76; *P* < 0.001) and pathological N positive disease (SHR 3.36, 95% CI 1.76–3.45; *P* < 0.001) were independently associated with CSM.

Finally, OM was significantly correlated to age at RC (HR 1.04, 95% CI 1.01–1.08; *P* = 0.001), preoperative NLR of >3 (HR 1.38, 95% CI 1.01–1.77; *P* = 0.01), hypoalbuminaemia (HR 0.63, 95% CI 0.49–0.92; *P* = 0.001), NOC disease (HR 1.49, 95% CI 1.19–5.76; *P* < 0.001) and lymph nodes involvement (HR 3.36, 95% CI 1.75–3.44; *P* < 0.001).

The discrimination of a standard multivariable model for recurrence (including age, ECOG PS score, organ-confined disease, concomitant carcinoma *in situ* (CIS), lymph node involvement and LVI increased from 69.4% to 72.1% and to 73.0% on inclusion of mGPS and of mGPS and fibrinogen, respectively. Similarly, the discrimination of the multivariable model for CSM increased from 71.3% to 73.1% and to 73.9% on inclusion of mGPS and of mGPS and fibrinogen, respectively.

## Discussion

Systemic inflammatory response plays a prominent role in the prognosis of oncological patients and accumulating evidence shows the critical role of the inflammatory process in mediating processes associated with the promotion of proliferation, angiogenesis and with invasion and metastasis [14]. Thus, systemic inflammation markers have been extensively investigated to develop prognostic models that could help the clinical decision-making process in oncology leading to the most suitable treatment option [15].

In the present study, we assessed the role of fibrinogen, mGPS, NLR, HDL and total cholesterol to predict long-term oncological outcomes in a selected elderly population treated with open RC for UBC. We found that in this population mGPS (1 and 2 vs 0) and higher fibrinogen values at baseline were independent predictors of major postoperative complications, disease recurrence, and CSM. A preoperative NLR of >3 and lower albumin were significantly associated with worse OM.

CRP is a well-known acute-phase reactant that is significantly elevated in oncological patients with poor prognosis [16] or metastatic disease [17]. Expanding on the utility of a single biomarker, the mGPS, calculated using CRP and albumin levels, has been identified as a meaningful marker of systemic inflammation. The rationale behind the influence of mGPS on oncological outcomes stands on the effects of systemic inflammation and progressive nutritional decline associated with advanced cancer and consequently a worse oncological outcome [18].

In 2015, Ferro *et al*. [19] first reported that mGPS was a valuable biological parameter to predict recurrence in a series of 1037 patients treated with RC. In a retrospective study involving 310 consecutive patients with clinical N0M0 UBC treated with RC, the mGPS was found to be an independent predictor of NOC disease after adjusting for clinical T-stage, LVI and abnormal radiological imaging [8]. Furthermore, it was also found that the mGPS was independently associated with intravesical recurrence after radical nephroureterectomy (RNU) in patients with upper urinary tract urothelial carcinoma (UTUC) [20].

Plasma fibrinogen is another acute reactive protein mainly synthesised by hepatocytes, and with an active role on clot formation, wound healing, and systemic inflammatory response. Hyperfibrinogenaemia has been associated with tumour progression and metastasis in lung [21], hepatic [22] and ovarian [23] cancer. Despite the mechanisms interplaying between cancer cells and fibrinogen remains unknown, it has been observed that interleukin-6, produced by lung cancer cells, induced fibrinogen secretion [24], while *in vitro* tests showed that cancer cells also synthesise fibrinogen promoting tumour cell growth and angiogenesis due to the interaction with other fibroblast and vascular endothelial growth factors [25].

The plasma fibrinogen level has been assessed in several retrospective series of patients treated with RNU for UTUC. These results have been evaluated in a systematic review and meta-analysis that concluded significant prognostic value to predict CSM after RNU [26]. Two other reviews highlighted the role of high levels of coagulation factors, including fibrinogen, in predicting poorer OS and CSS in the broad spectrum of urological malignancies [27,28]. Nevertheless, to our knowledge the impact of baseline fibrinogen on the surgical and oncological outcomes after RC has never been explored. Our present series represent the first attempt to specifically correlate elevated pretreatment fibrinogen levels with major complications, disease recurrence, OM and CSM in a cohort of elderly patients with UBC treated with RC. In detail, we found that the discrimination of a standard multivariable model (including age, ECOG PS score, organ-confined disease, concomitant CIS, lymph node involvement, LVI) increased from 69.4% to 73.0% to predict recurrence and from 71.3% to 73.9% to predict CSM by adding mGPS and fibrinogen.

In a retrospective study by D’Andrea *et al*. [29] involving 4335 patients undergoing RC for clinically non-metastatic UCB, the NLR independently improved the preoperative prediction of lymph node metastasis and survival outcomes. However, fibrinogen and mGPS were not investigated. Based on the most frequent value analysed in literature, the NLR threshold was set at >3. In our present study, values over this threshold were not statistically associated either with disease recurrence or with CSM. Nevertheless, it was independently correlated to OM. This may indicate that NLR represents a less accurate marker than mGPS and fibrinogen in the elderly population.

The absence of an enhanced recovery after surgery protocol in all patients in our retrospective database represents a further limitation of the study. Today, it is of paramount importance that the patient’s condition is optimal at the time of RC; indeed, the positive influence of a perioperative immune nutrition diet for reduced incidence of perioperative complications and re-admission has been recently demonstrated in a randomised clinical trial [30]. Fibrinogen in conjunction with CRP and albumin may potentially become a useful parameter not only for the evaluation of oncological outcomes, but also for the identification of a subgroup of patients that could benefit more from immunostimulatory therapy before RC.

Moreover, we considered advanced age as a surrogate of frailty; however, age can only partially capture frailty, while the use of a frailty index would have been more accurate [31]

Despite the several limitations of our present study, it must be noted that it was the first time that fibrinogen and mGPS were evaluated together in an elderly population with UBC amenable to treatment with RC. Our present results add evidence to the growing body of literature on the prognostic role of the systemic markers of inflammation. The simplicity and cost-effectiveness of their assessment are major advantages, although further clinical studies are needed to identify the importance of fibrinogen in the general population treated with RC.

## Conclusion

In a cohort of elderly patients with UBC treated with RC, several biomarkers of systemic inflammatory response seem to be correlated with perioperative and oncological outcomes. In particular, fibrinogen and mGPS appeared to be the most relevant prognostic measurements increasing the accuracy of clinicopathological preoperative models to predict major postoperative complications, recurrence and mortality.
